# Olanzapine Induces Adipogenesis and Glucose Uptake by Activating Glycolysis and Synergizing with the PI3K-AKT Pathway

**DOI:** 10.2174/1570159X22666240815120547

**Published:** 2024-08-15

**Authors:** Shen Li, Yun Fu, Wanyao Wang, Jiali Qiu, Yepei Huang, Xuemin Li, Ke Yang, Xiawen Yu, Yanyan Ma, Yuan Zhang, Miaomiao Zhang, Jie Li, Wei-Dong Li

**Affiliations:** 1 Laboratory of Biological Psychiatry, Institute of Mental Health, Tianjin Anding Hospital, Mental Health Center of Tianjin Medical University, Tianjin, 300222, China;; 2 Department of Genetics, College of Basic Medical Sciences, Tianjin Medical University, Tianjin, 300070, China;; 3 Fujian Maternity and Child Health Hospital College of Clinical Medicine for Obstetrics and Gynecology and Pediatrics, Fujian Medical University, Fuzhou, 350001, China

**Keywords:** Olanzapine, adipocyte, insulin resistance, lipolysis, glycolysis, PI3K/AKT

## Abstract

**Background:**

Administration of olanzapine (OLA) is closely associated with obesity and glycolipid abnormalities in patients with schizophrenia (SCZ), although the exact molecular mechanisms remain elusive.

**Objective:**

We conducted comprehensive animal and molecular experiments to elucidate the mechanisms underlying OLA-induced weight gain.

**Methods:**

We investigated the mechanisms of OLA-induced adipogenesis and lipid storage by employing a real-time ATP production rate assay, glucose uptake test, and reactive oxygen species (ROS) detection in 3T3-L1 cells and AMSCs. Rodent models were treated with OLA using various intervention durations, dietary patterns (normal diets/western diets), and drug doses. We assessed body weight, epididymal and liver fat levels, and metabolic markers in both male and female mice.

**Results:**

OLA accelerates adipogenesis by directly activating glycolysis and its downstream PI3K signaling pathway in differentiated adipocytes. OLA promotes glucose uptake in differentiated 3T3-L1 preadipocytes. In mouse models with normal glycolipid metabolism, OLA administration failed to increase food intake and weight gain despite elevated GAPDH expression, a marker related to glycolysis and PI3K-AKT. This supports the notion that glycolysis plays a significant role in OLA-induced metabolic dysfunction.

**Conclusion:**

OLA induces glycolysis and activates the downstream PI3K-AKT signaling pathway, thereby promoting adipogenesis.

## 1 INTRODUCTION

Antipsychotic drugs are the mainstay of treatment for schizophrenia (SCZ) [1]. In recent years, antipsychotic drugs have been increasingly prescribed for other mental disorders, even off-label indications [2]. Compared with first-generation antipsychotic drugs (FGAs), second-generation antipsychotics (SGAs) demonstrate superior curative effects on the positive and negative symptoms of SCZ [3, 4]. Unfortunately, SGAs could cause metabolic side effects, including weight gain, hyperglycemia, dyslipidemia, and cardiovascular diseases. Olanzapine (OLA) and clozapine (CLO) exhibit the worst profiles in terms of metabolic abnormalities [5]. However, patients with severe psychosis tend to have inherent risks of developing obesity and metabolic syndrome (MetS) [6, 7], as well as various hormonal disturbances and unhealthy lifestyles [8]. SGAs cannot be ignored in promoting weight gain and metabolic abnormalities in psychosis patients [9]. These side effects have become the predominant cause of premature death in SCZ patients [10]. Therefore, it is urgent to decipher the mechanisms of SGA-induced weight gain.

Numerous studies have focused on the effects of SGAs on the central nervous system (CNS), such as modulation of energy homeostasis through central serotoninergic 2A and 2C, adrenergic α, and dopaminergic D2 receptors [11], as well as stimulation of the appetite through central histaminergic pathways [12]. More recently, studies have shown that SGAs might act directly on adipose tissues to change body energy homeostasis [13]. Adipose tissue dysfunction leads to abnormal production and secretion of adipokines, which are known to contribute to the pathogenesis of obesity and MetS [14]. Among adipose-derived adipokines, adiponectin (APN) and leptin (LEP) are two major players in SGA-induced weight gain/metabolic disturbances [15, 16]. We have previously found that *ADIPOQ* gene (a gene encoding APN) polymorphisms were associated with SGAs-induced increases in body weight and waist-to-hip ratio [17]. Although the direct effects of SGAs on adipose tissues have been documented, little is known about the molecular and cellular mechanisms responsible for the changes in weight and metabolism.

Weight gain and metabolic abnormalities caused by OLA and other antipsychotics are important concerns. Decades of studies have shown that dozens of genes contribute to susceptibility to antipsychotics-induced weight gain (AIWG) [17-19]. However, several key issues remain unclear. Although AIWG is common in SCZ patients, equivocal and even controversial results have been observed in mice [20, 21]. On the other hand, many studies have focused on appetite and food intake, while the direct effect of OLA on adipocyte differentiation/adipogenesis has been poorly investigated. Although the expression of the transcription factor CCAAT/enhancer-binding proteins (C/EBPs), proliferator-activated receptor gamma (PPARG), and sterol regulatory element-binding protein (SREBP) pathway genes were found to increase in OLA-treated adipocytes [13, 22, 23], it is hard to interpret those changes as reasons rather than results of adipogenesis. Little is known about how AIWG (*i.e*., adipogenesis) was initiated, which key genes or pathways were induced by OLA, and how these pathways were synchronized.

In this study, we carried out a full-spectrum investigation to decipher the effects of OLA on adipocytes and animal models. Furthermore, we examined the effects of OLA on adipogenesis and lipid storage in both preadipocytes and mouse models.

## 2 MATERIALS AND METHODS

### 2.1 Drugs, Adipose Tissue Mesenchymal Stem Cell and Preadipocyte Differentiation

**Fig. (1):**
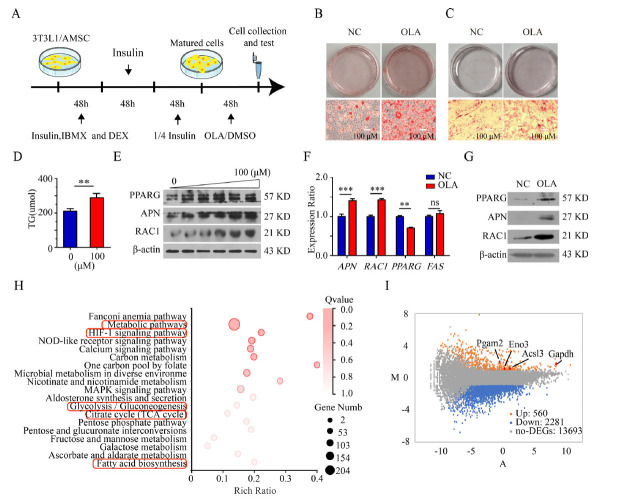
Effect of olanzapine (OLA) on triglyceride accumulation and mRNA and protein expression in 3T3-L1 and adipose tissue-derived canine mesenchymal stem cells (AMSCs). (**A**) a workflow of this research. (**B**) Oil red O (ORO) staining of differentiated 3T3-L1 cells (N=3). (**C**) ORO staining of differentiated AMSCs after OLA treatment (N=3). (**D**) Triglyceride accumulation changes in differentiated 3T3-L1 cells during 72 hours of treatment with OLA (N=3). (**E**) Western blotting analysis of proliferator-activated receptor gamma (PPARG), adiponectin (APN) and Ras-related C3 botulinum toxin substrate 1 (RAC1) in differentiated 3T3-L1 cells that were treated with OLA (0-100 μM) (N=3). (**F**) Relative gene transcription levels of PPARG, APN, RAC1 and fatty acid synthase (FAS) in differentiated 3T3-L1 cells (N=3). (**G**) Western blotting analysis of PPARG, APN, RAC1 in differentiated AMSCs after OLA treatment (N=3). (**H**) Kyoto Encyclopedia of Genes and Genomes (KEGG) pathway enrichment analysis of differentially expressed genes (N=2). (**I**) MA plot of up- and downregulated genes following OLA treatment in 3T3-L1 cells. Upregulated gene in Glycolysis / Gluconeogenesis pathway: Pgam2: Phosphoglycerate mutase 2. Eno3: Enolase3. Acsl3: Long-chain-fatty-acid-CoA ligase 3. GAPDH: Glyceraldehyde-3-phosphate dehydrogenase. Values are expressed as the mean ± SD, *P < 0.05, **P < 0.01, ***P < 0.001, ns, no significant difference. SD: Standard deviation. Ruler: 50 μm.

Olanzapine was dissolved in stock solutions of dimethyl sulfoxide (DMSO) (0.01 M) and stored at -20℃ until use. For an experiment, olanzapine solutions were diluted in PBS. The final concentration of DMSO in the vehicle was 1% for cell culture experiments and 2% for animal experiments. 3T3-L1 embryonic fibroblasts and adipose tissue mesenchymal stem cells (AMSCs) are used in this study, which are commonly used models for studying adipocyte differentiation *in vitro*. AMSCs were isolated as previously described [24]. Briefly, adipose tissue dissected from the medial extremity of 6-week-old mice (C57BL/6J) was washed 3 times in PBS solution containing 2% penicillin-streptomycin and cut into fine fragments. Furthermore, the fragments were dispersed into single cells using collagenase 1 for 1 hour, followed by culture in Dulbecco's modified Eagle medium (DMEM) containing 20% fetal bovine serum, 1% L-glutamine and 1% penicillin-streptomycin for approximately 4 days. Preadipocytes 3T3-L1 (BeNa Culture Collection, China) and AMSCs were maintained in a culture medium consisting of 89% DMEM, 10% fetal bovine serum and 1% penicillin-streptomycin. For cell differentiation (Fig. **1A**), cells were sequentially treated with MDI (1 μM dexamethasone, 0.5 mM isobutyl-methylxanthine and 10 μg/ml insulin) for 48 hours, 10 μg/ml insulin for another 48 hours and 2.5 μg/ml insulin for the last 48 hours. Differentiated 3T3-L1 cells and AMSCs were treated with DMSO or 100 μM OLA for 48 hours. All experiment reagents as shown in Table **S1**. Preadipocytes (3T3-L1) and AMSCs were treated with OLA (100 μM) for 2 days, 3T3-L1 cells and AMSCs were stained with Oil red O, and cells were harvested for triglyceride quantification (Comin, Suzhou). Western blotting was performed to detect the expression levels of adipogenesis markers.

Differentiated 3T3-L1 cells and AMSCs were treated with DMSO or 100 μM OLA for 48 hours. 3T3-L1 cells and AMSCs were stained with Oil red O, and cells were harvested for triglyceride quantification (Comin, Suzhou). Western blotting was performed to detect the expression levels of adipogenesis markers. Meanwhile, a real-time ATP production rate assay, a glucose uptake test, ROS detections in OLA-treated 3T3-L1 cells, and small interfering RNA were performed to study the effect of OLA on glycolysis and the PI3K-Akt pathway. Related information is provided in the Supplementary Tables **S2**, **S3** & **S4**.

### 2.2 Total Protein Extraction and Immunoblots

Western blot analysis was performed as previously described [25]. 3T3-L1 and AMSC cells were lysed in lysis buffer (50 mM Tris, pH 7.6, 150 mM NaCl and 1% Triton X-100) containing protease inhibitors and PMSF for 30 min and then centrifuged at 12,000 rpm for 15 min. The extraction method of total protein from mouse adipose tissue refers to the adipose tissue extraction kit (HR0049, Biolab, CHN). Briefly, after fully lysing the adipose tissue cut with scissors for 30 minutes with the lysis solution, the whole protein solution was transferred to the filter tube and centrifuged at 10000g for 10 minutes to obtain the adipose tissue whole protein extract after which the supernatants were harvested and boiled with loading buffer for five minutes. After sample preparation, equal amounts of sample protein were loaded onto an SDS-PAGE gel and transferred onto a PVDF membrane. Then, the membrane was blocked for 1 hour at room temperature and incubated overnight at 4°C with the following primary antibodies: anti-β-actin (cat#bs10966R, 1:5000, Bioss, CHN), anti-RAC1 (cat#ab33186, 1:1000, Abclonal, CHN), anti-PPARG (cat#bsm33436m, 1:1000, Bioss, CHN), anti-APN (cat#bs0471R, 1:1000, Bioss, CHN), anti-GAPDH (cat#bs2188R, 1:5000, Bioss, CHN), anti-FOXO1 (cat#2880T, 1:1000, CST, USA), anti-P-FOXO1 (cat#9461T, 1:1000, CST, USA), anti-PI3K-P110A (cat#4249T, 1:1000, CST, USA), anti-PDK1 (cat#A8930, 1:1000, Abclonal, CHN), anti-P-PDK1(cat#3438T, 1:1000, CST, USA), anti-AKT (cat#9272S, 1:1000, CST, USA), anti-P-AKT (cat#13038P, 1:1000, CST, USA), anti-GLUT1 (cat#A6982, 1:1000, Abclonal, CHN), anti-GLUT4 (cat#A7637, 1:1000, Abclonal, CHN), anti-HIF1A (1:1000, CST, USA). After washing with TBST, membranes were incubated with goat anti-mouse or goat anti-rabbit HRP-conjugated secondary antibody (cat#S0001&S0002, 1:10000, Affinity, USA) for 2 hours at room temperature and detected by ECL. The results were normalized using β-actin as an internal control and quantified by densitometry using Image J software (Image J 1.5, NIH, USA). Each experiment was performed in triplicate.

### 2.3 Oil Red-O Staining and Triglyceride Quantification

Cells were washed twice with PBS and fixed in 4% paraformaldehyde solution for 30 min at room temperature, followed by staining with freshly diluted 0.5% Oil red O for 15 min. The 0.5% Oil red O dye solution is made up of 0.5 g Oil red O powder dissolved in a solution containing 60% isopropyl alcohol and 40% water, shaken at room temperature for 1 hour and filtered to obtain a clear solution. Intracellular triglyceride was tested by the Tissue triglyceride test kit according to the kit instructions. Briefly, cells were washed twice with PBS and digestion into single cells using trypsin, then 3x10^6^ cells from each group were taken and lysed with cell lysate buffer for 30 minutes at 4℃, after centrifuging at 12,000 rpm (room temperature) for 15 minutes, the supernatant was used for the quantitative detection of triglyceride.

### 2.4 cDNA Synthesis and Real-time PCR

Total RNA was extracted from 3T3-L1 cells by the TRIzol extraction method. 1 mg of total RNA of each sample was reverse transcribed with a cDNA Synthesis Kit (Biomake). Real-time PCR amplification was performed using a 7500 Real-Time PCR system (Applied Biosystems, USA). The mRNA expression level was normalized to the expression level of the housekeeping gene β-actin. Relative gene expression levels were calculated using 2-△△Ct. Each experiment was performed in triplicate. The primers are shown in Table **S3**.

### 2.5 RNA-sequencing and Data Analyses

Two replicated RNA-seq libraries were prepared from DMSO and OLAN (100μM) treated 3T3-L1 cells (differentiated cells). A total of the four libraries were sequenced separately using the BGISEQ-500 sequencer (BGI, Shenzhen, CHINA). For each RNA sample, the cells were collected from three replicates and pooled together after RNA extraction. Raw sequencing reads were cleaned by removing adaptor sequences, reads containing poly-N sequences, and low-quality reads. After data were mapped, normalization was performed, and then FPKMs (fragments per kilobase per million mapped reads) were calculated using the DC.TOM platform. The significant levels of terms and pathways were corrected by Q value with a rigorous threshold (Q value < = 0.05) by Bonferroni. The KEGG pathway enrichment was carried out by the GSEA (gene-set enrichment analysis) algorithm, pathways were plotted by Q-values and enrichment scores (rich values, Fig. **1H**).

### 2.6 Real-time ATP Production Rate Assay, Mitochondria Stress Test Assay, Glucose Uptake, and ROS Detection in OLA-treated 3T3-L1 Cells

The real-time ATP production rate assay was used to analyze the rate and pathway of cellular ATP production. First, differentiated 3T3-L1 cells (treated with DMSO or OLA (100 μM) for 48 h) were suspended in trypsin solution (1.25 g/L), seeded in Seahorse XF24 cell culture microplates and incubated overnight. Meanwhile, the detection probes were incubated with an XF calibrant solution for at least 4 h. After culturing for 12 hours, the cells were washed twice with seahorse serum-free DMEM (pH 7.4, 10 mM glucose, 1 mM pyruvate, 2 mM glutamine). Then, the detection solution was added to the cells and incubated at 37°C for 60 min without CO_2_. Finally, the probe plate was placed on top of the cell culture plate, and the ATP generation rate of cells was detected by a Seahorse XF Real-Time ATP Rate Assay Kit (Agilent Technologies, Co., USA) using Seahorse XFe24. The ATP production rate was detected. The Mitochondria stress test assay was performed similarly to the real-time ATP production rate assay, all procedures were according to the mitochondria stress test assay user guide and the FCCP was used in 1.25 μM as the final concentration. The glucose uptake assay was used to detect the effect of drugs on the cellular uptake rate of glucose. Glucose uptake was detected by the 2-NBDG Glucose Uptake Assay kit (cell-based) (BioVision, Brugg, Switzerland). After preadipocytes were differentiated and treated with OLA (100 μM) or DMSO for 48 hours, cells were suspended in trypsin and seeded into a 24-well tissue culture plate. After 12 hours, regular culture was removed, and cells were treated with test compound or vehicle in 400 μl cell culture medium with 0.5% FBS and incubated at 37°C with 5% CO_2_ for 1 hour. After incubation, cells were collected from the plate and suspended in 400 µl of 1X Analysis Buffer, which was analyzed on a flow cytometer (488 nm excitation laser) (Beyotime, Nanjing). The total intracellular ROS production level was determined by a reactive oxygen species assay kit. Briefly, cells were washed with PBS and incubated with 10 μM DCFH-DA at 37°C for 30 min, followed by detection by fluorescence microscopy and flow cytometry (Fig. **S2B**).

### 2.7 Small Interfering RNA and Inhibitors of Glycolysis and the PI3K-Akt Pathway

3T3-L1 preadipocytes grown in 60 mm plates were treated with 100 nM small interfering RNA (siRNA) targeting GAPDH (glyceraldehyde-3-phosphate dehydrogenase) and control RNA (Syniobio Technologies, Suzhou). The SiGAPDH primers are shown in Table **S2**. The Lipofectamine RNAiMAX (Invitrogen, Co., USA)-mediated siRNA transfection method was used. Specifically, Lipofectamine was diluted using Opti-MEM serum-free media and was mixed and incubated with siRNA for 15 minutes. After that, the mixture was added to cells, which were approximately 70–90% confluent. Ascorbic acid (0.5 mg/L for 48 h) and PI3KIN1 (25 μM for 48 h) were used to inhibit GAPDH and PI3K enzyme activity, respectively. Real-time PCR was performed to detect adiponectin, Ras-related C3 botulinum toxin substrate 1 (RAC1), peroxisome proliferator-activated receptor gamma (PPARG), fatty acid synthase (FAS), and glyceraldehyde-3-phosphate dehydrogenase (GAPDH).

### 2.8 Animal Experiments

We carried out animal experiments on OLA-induced weight gain in mouse and rat models with different metabolic backgrounds and diets. Adult male and female C57BL/J mice, Sprague Dawley rats, and KK/Upj-ay mice (6-8 weeks, Huafukang, China) were used in this study.

After 5 days of acclimatization and 5 days of gavage adaptation, the 8-week-old adult male and female rodents were divided randomly into 9 groups (5-10 per group) as follows (Table **S4**). We carried out *in vivo* drug tests in the animal through oral administration, considering five variables, including medication time, medication dose, sex, genetic background, and diet. The medication time included short-term medication for 2 weeks and long-term medication for 3-6 months. The dosage of our medicine was divided into two groups: the normal dosage and the high dose according to the conversion of body surface area, and the normal dosage was the main set. To exclude the influence of genetic backgrounds, we selected three animal strains with different genetic backgrounds, including C57BL/6J (susceptible to diet-induced obesity), Sprague Dawley (susceptible to sex hormones) Rats and KK/Upj-Ay (overeating, hyperglycemia, insulin resistance). Considering dietary factors, in addition to maintaining a normal diet structure, we specifically added a Western diet structure model, which contains 20% protein, 50% carbohydrate and 21% fat. It is a commonly used animal model diet to imitate the Western diet structure.

### 2.9 Body Weights, Food Intake, Epididymal and Liver Fats

For rodents’ food intake and body weight data, we appropriately adjusted the collection time according to the experimental situation. We recorded the food intake of rodents daily and collected weight data at intervals of up to four days during the entire experiment. To weigh the liver and adipose tissue around the epididymis and ovary, when the experimental period was over, the mice and rats were anesthetized with isoflurane (500 ml/min) for one minute, and blood was collected from the eyes. After confirming the death of the mice and rats, the mice and rats were dissected, and their ovarian and epididymal fat and liver were quickly separated for weighing.

### 2.10 Metabolism and Biochemical Evaluations in Mice

For the fasting glucose tolerance test in mice, we first treated the mice with overnight fasting for 12 hours. Before the start of the experiment, we used a sterile instrument to cut off the distal end of the tail by approximately 0.5 cm. After taking blood from the tail to measure fasting blood glucose, each mouse was intraperitoneally injected with 0.5 mg/kg glucose solution, and blood was taken at regular intervals to measure blood glucose. Blood metabolism biochemical markers (fasting glucose, high-density lipoprotein, low-density lipoprotein, *etc*.) were detected by an assay kit (Nanjing Jiancheng, Nanjing, China) (Table **S1**), and all experimental procedures were consistent with the kit instructions.

### 2.11 Liver Sections and Oil Red-O Staining

After the mice were sacrificed by neck dissection, the liver tissue of about 0.5 cm in diameter was quickly cut with a blade and fixed with OCT embedding glue and then sliced using a cryostat (LECA CM9150) with a thickness of 20 microns. The cut tissue section was placed on a glass slide, fixed with formaldehyde for 5 minutes, and washed with running water for about 20 seconds and then the tissue section was stained with oil red O dye solution for 15 minutes, followed by being washed with running water for about 1 minute. The resulting stained section was photographed with an imaging microscope.

### 2.12 Statistical Analysis

Data in the figures are presented as the mean ± standard deviation (SD). Statistical analyses were carried out using SPSS version 22.0 (IBM Corporation, Armonk, New York, USA) and GraphPad Prism version 8.0 (GraphPad Software Inc, La Jolla, USA). Significance was accepted at *P* < 0.05.

## 3 RESULTS

### 3.1 OLA Promotes Triglyceride Storage in Adipocytes but not in Preadipocytes

We examined the effects of OLA on triglyceride storage. Specifically, 3T3-L1 cells were induced to undergo adipogenic differentiation (using differentiation medium) for 6 days, followed by OLA treatment for 48 hours (Fig. **1A**). Oil red O staining showed that OLA treatment caused a significant increase in lipid production (Fig. **1B**). To validate these observations, we isolated primary AMSCs from the adipose tissue of 6-week-old C57/BL6 mice and measured them by oil red O staining, and we obtained the same results (Fig. **1C**). Furthermore, the intracellular levels of triglycerides in differentiated 3T3-L1 cells were quantitatively measured, indicating a 50% increase in triglyceride accumulation in the experimental group (*p* < 0.01) (Fig. **1D**). APN, Ras-related C3 botulinum toxin substrate 1 (RAC1) and PPARG are important molecular markers of adipocyte differentiation and triglyceride storage [26-28]. Thus, we detected the protein expression levels (Fig. **1E** and Fig. **S1A**, **B** & **C**) and mRNA levels (Fig. **1F**) of adipogenesis markers and found that the expression levels of APN, RAC1, and PPARG increased under OLA treatment in a dose-dependent manner in differentiated 3T3-L1 cells. In the cultured AMSCs, the protein expression of PPARG, APN, and RAC1 increased after OLA treatment (Fig. **1G** and Fig. **S1D**).

To investigate the effects of OLA on adipocyte differentiation, 3T3-L1 cells and AMSCs were cultured with OLA but without differentiation medium (MDI) for 7 days. Oil red O staining showed that OLA did not affect triglyceride accumulation in the primary cultured adipocytes (Fig. **S1E** & **F**). Related markers of adipocyte differentiation, including APN, Rac1 and PPARG, did not change significantly in primary cultured adipocytes. Taken together, OLA promoted triglyceride storage in differentiated adipocytes.

### 3.2 OLA Causes Cellular Metabolic Disorders and Activates Genes in the Glycolysis Pathway

To determine the molecular mechanisms of triglyceride storage in response to OLA treatment, adipocytes treated with DMSO and OLA were tested by RNA sequencing (RNA-seq). Compared to the DMSO treatment group, OLA treatment caused 580 upregulated genes and 2281 downregulated genes. In the analysis of the Kyoto Encyclopedia of Genes and Genomes (KEGG), these up-regulated mRNAs were enriched. The most enriched molecular functions were related to metabolism, glycolysis/gluconeogenesis, fatty acid biosynthesis, and the citrate cycle (TCA cycle) (Fig. **1H**). We found that obvious upregulation of Phosphoglycerate mutase 2, Enolase3, Long-chain-fatty-acid--CoA ligase 3, and Glyceraldehyde-3-phosphate dehydrogenase (GAPDH) transcription in the glycolysis pathway (Fig. **1I**).

### 3.3 OLA Facilitates Glucose Uptake and Promotes Glycolysis

**Fig. (2):**
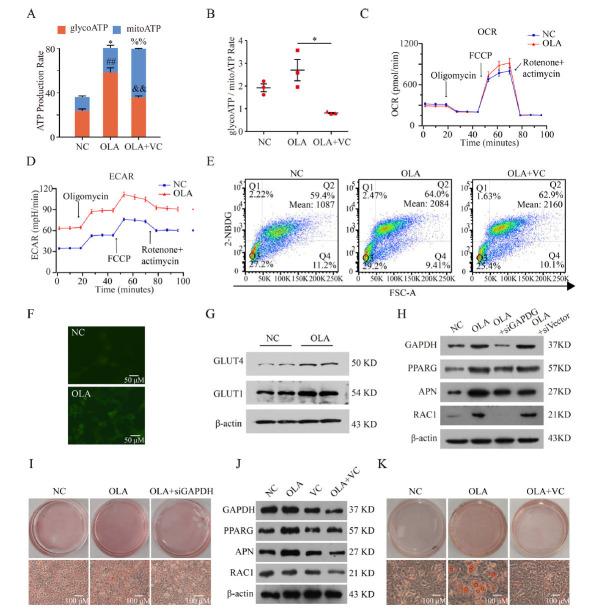
OLA promotes differentiated 3T3-L1 glucose uptake and increases the rate of glycolysis to produce ATP. (**A**) Rate of intracellular mitochondria and glycolysis to ATP after treatment with OLA and vitamin C (VC). ##: OLA glycolysis ATP production rate compared with NC glycolysis ATP production rate. *P* < 0.01. *: ATP production rate in OLA mitochondria compared with the ATP production rate in NC mitochondria. *P* < 0.05. &&: OLA glycolysis ATP production rate compared with NC glycolysis ATP production rate. *P* < 0.01. %%: OLA mitochondria ATP production rate compared with NC mitochondria ATP production rate. *P* < 0.01. (**B**) The ratio of the glycolytic ATP production rate to the mitochondrial ATP production rate (N=3). (**C**) O_2_ consumption rate (OCR) after treatment with OLA. (**D**) Extracellular acidification rate (ECAR) after treatment with OLA. (**E**) Glucose uptake in 3T3-L1 cells exposed to DMSO, OLA, OLA plus VC (0.1 g/L). (**F**) Glucose uptake under fluorescence microscope. (**G**) Western blotting analysis of GLUT1 and GLUT4 in differentiated 3T3-L1 cells treated with OLA (N=3). (**H**) Western blotting analysis of GAPDH, PPARG, APN and RAC1 in untreated control, OLA, and OLA+siGAPDH, and OLA+siVector (scrambled siRNA control) treated 3T3-L1 cells (N=3). (**I**) Representative image of 3T3-L1 ORO staining after the interference of GAPDH expression. (**J**) Relative gene transcription of adipocyte markers and the cell metabolism markers PPARG, APN and RAC1 in untreated control, OLA, VC, and OLA+VC treated 3T3-L1 cells (N=3). (**K**) Representative image of 3T3-L1 ORO staining after VC inhibition. Values are expressed as the mean ± SD, *P < 0.05, **P < 0.01, ***P < 0.001, ns, no significant difference. Ruler: 50 μm.

According to the RNA-seq analysis, we further investigated whether OLA induced triglyceride accumulation by increasing glycolysis. To identify the major glucometabolic pathway, the ATP synthase rate was detected by the Seahorse assay. We measured the total ATP production rate, glycolytic ATP production rate, and mitochondrial ATP production rate in DMSO- and OLA-treated 3T3-L1 cells. The ATP synthase rate, especially the glycolytic ATP production rate, was significantly increased in mature adipocytes after OLA treatment (Figs. **2A** & **B**), indicating that glycolysis and corresponding ATP production were enhanced under OLA treatment. In order to evaluate the effects of OLA on the mitochondria of differentiated 3T3-L1 cells, we performed the mitochondria stress assay, and the results showed that OLA did not alter the basal respiration of differentiated 3T3-L1 cells except for the maximal respiration measured after the injection of the uncoupling agent FCCP (Fig. **2C**), implying that OLA enhanced the maximal respiration of differentiated 3T3-L1 cells. Besides, we found that the extracellular acidification rate (ECAR) of differentiated 3T3-L1 cells was increased after OLA treatment (Fig. **2D**).

Furthermore, we performed a 2-NBDG uptake experiment and found that OLA increased cellular glucose uptake by nearly 2-fold (Fig. **2E** & **F**, left and middle and Fig. **S1G**). In addition, the expression levels of GLUT1 and GLUT4 were increased (Fig. **2G** and Fig. **S1H**). This result indicated that OLA facilitates glucose uptake and enhances the activity of glycolysis.

### 3.4 OLA Promotes Triglyceride Storage by Enhancing the Glycolysis Pathway

To investigate the effects of glycolysis on triglyceride accumulation under OLA treatment, GAPDH (a rate-limiting enzyme in glycolysis) was detected and found to be upregulated at both transcription and protein levels (Fig. **S1I** & **J**). The downregulation of GAPDH function was performed by siRNA or ascorbic acid (vitamin C, VC). VC is widely used as a glycolysis inhibitor targeting GAPDH to study the role of glycolysis in cancer treatment [29]. The transcription and protein expression levels of adipogenesis-related markers, including PPARG, APN, and RAC1, were also decreased by GAPDH-siRNA (Fig. **2H** and Fig. **S1K**) and VC, indicating that elimination of OLA-induced lipogenesis by inhibiting glycolysis (Fig. **2J** and Fig. **S1L**). Oil red O staining showed that the inhibition of GAPDH attenuated OLA-induced lipid accumulation (Fig. **2I** & **K**). These data indicate that enhanced glycolysis is the main cause of cellular triglyceride accumulation promoted by OLA treatment.

### 3.5 Reactive Oxygen Species (ROS) are Elevated, and the PI3K-AKT Pathway is Activated by Olanzapine Treatment

**Fig. (3):**
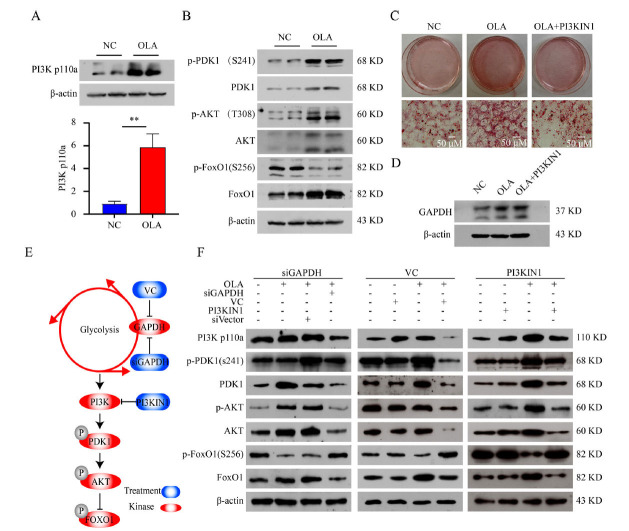
OLA increased adipogenesis in differentiated 3T3-L1 cells through glycolysis to activate the PI3K-AKT axis. (**A**) Intracellular expression of PI3K after treatment with DMSO or OLA. OLA has raised the PI3K expression (N=3). (**B**) Phosphorylation levels of the PI3K-AKT axis-related proteins (N=3). (**C**) Oil red O staining of 3T3-L1 cells after inhibition with PI3KIN1. (**D**) Intracellular expression of GAPDH after treatment with DMSO or olanzapine and PI3KIN1 (N=3). (**E**) Schematic of glycolysis and the PI3K-Akt signaling pathway. (**F**) After inhibiting GAPDH or PI3K, immunoblots of PI3K-AKT axis-related protein expression and phosphorylation levels (N=3). Values are expressed as the mean ± SD, *P < 0.05, **P < 0.01, ***P < 0.001, ns, no significant difference.

To further characterize the cellular metabolism in 3T3L1 cells, the levels of reactive oxygen species (ROS) were detected using fluorescence microscopy and flow cytometry. As shown in Supplementary Fig. **S2A** and **B**, the ROS levels increased after OLA treatments. To verify whether PI3K and downstream proteins could be activated by ROS, and OLA might activate the ROS-PI3K axis through glycolysis activation, we tested the expression levels and phosphorylation levels of proteins in the PI3K-AKT pathway by Western blots. We found that PI3K, PDK1, AKT, and FOXO1 were elevated in response to OLA treatment, and the phosphorylation levels of PDK1 and AKT were also increased, while the phosphorylation levels of FOXO1 were decreased (Figs. **3A** & **B** and Fig. **S1M**).

### 3.6 OLA Promotes Triglyceride Storage by Activating the Glycolysis-PI3K-AKT Axis

Immunoblotting results revealed that the PI3K pathway was activated by OLA treatment. We further used PI3KIN1 (a PI3K inhibitor) to downregulate PI3K activity and examined the accumulation of cellular triglycerides. As expected, triglyceride accumulation was alleviated by PI3KIN1 treatment, indicating that PI3K plays an essential role in lipogenesis (Fig. **3C**). To clarify the regulatory network of triglyceride accumulation by glycolysis and PI3K-AKT axis exposed to OLA treatment, the glycolysis pathway was inhibited with siRNA or VC after OLA treatment (Fig. **3D** & **E**). As shown in Fig. **3F** and Fig. **S1N**, O & P, OLA-induced activation of the PI3K-AKT pathway was rescued by glycolysis inhibition and PI3KIN1 treatment. Taken together, OLA promotes triglyceride storage in adipocytes by enhancing the glycolysis pathway and further activating the downstream PI3K-AKT axis.

### 3.7 OLA did not cause Weight Gain or Metabolic Abnormalities in Animal Models

**Fig. (4):**
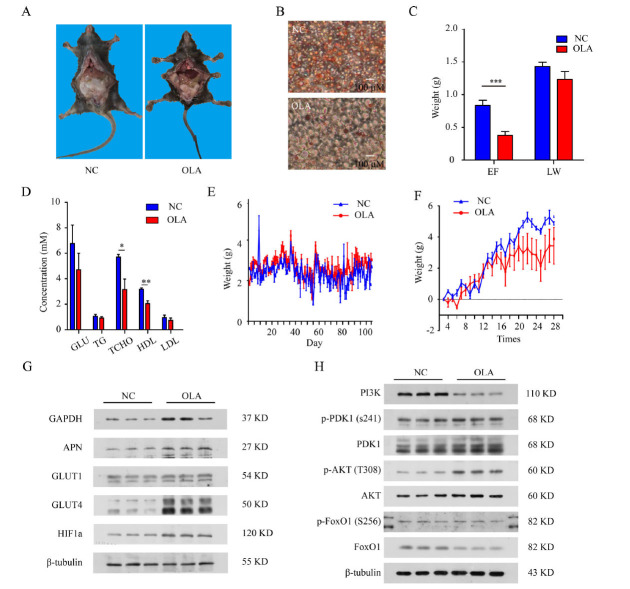
OLA did not increase mouse weight or metabolic disorders but increased glycolysis and glucose transport. C57BL/6J male mouse treated with Saline or OLA 3 month. (**A**) Mouse anatomy, compared with gavage with saline (left) and gavage with OLA, 3 mg/kg/day (right). (**B**) Mouse hepatic tissue with Oil red O staining. Under: Mice with OLA. Down: Mice with saline. (**C**) Mouse epididymal fat (EF) and liver fat weight (LW) (control = 4, OLA = 5). (**D**) Glucose and lipid profiles in mouse serum, including fasting glucose (GLU), triglycerides (TG), total cholesterol (TCHO), high-density lipoprotein (HDL), and low-density lipoprotein (LDL). (**E**) Changes in food intake during the experiment. (**F**) Changes in net weight gain of mice during the experiment (measured every 4 days). (**G**) Immunoblot analysis of GAPDH, adiponectin (APN), GLUT1, GLUT4, and HIF1α in mouse adipose tissues. (**H**) Immunoblot analysis of PI3K-AKT axis-related proteins in mouse adipose tissue (N=3). Values are expressed as the mean ± SD, *P < 0.05, **P < 0.01, ***P < 0.001, ns, no significant difference. Ruler: 100 μm.

To verify the relationship between the risk of obesity and OLA, healthy male mice (C57BL/6J) were treated with OLA treatment by gavage administration for 3 months. Unexpectedly, mice exposed to OLA showed little weight gain and even a reduction in abdominal fat (Fig. **4A**). The OLA-treated mice had less liver fat accumulation than those in the control group (Fig. **4B**). Then, we checked the weight of liver and epididymal fat in mice and found that epididymal fat decreased in the OLA exposure group, but the liver weight did not change significantly (Fig. **4C**). To investigate whether OLA affects glucose and lipid metabolism in mice, we examined relevant biochemical indices. Both serum high-density lipoprotein (HDL) and total cholesterol were lower in the mice taking OLA than those in the control group (Fig. **4D**). In fact, the daily food intake and net weight gain of mice did not show significant differences between the experimental and control groups. However, after OLA administration, mice in the experimental group showed a greater variance in body weight than those in the control group (Fig. **4E** & **F**). We also detected the expression of glycolysis- and glucose transport-related proteins, such as GAPDH and glucose transporters, in adipose tissue, and we obtained similar results with 3T3-L1 cells (Fig. **4G** and Fig. **S1Q**). Interestingly, we found that OLA exposure led to the activation of GAPDH and glucose transport proteins but not PI3K-AKT pathway proteins, which were even inhibited (Fig. **4H** and Fig. **S1R**). Furthermore, additional experiments with gavage administration were carried out, and showed that the food intake, body weight, blood glucose, and abdominal fat of mice did not change significantly under short-term OLA exposure (Table **S4**, groups 1, 2, 3 and 4). Body weight had a downward trend after OLA exposure (Fig. **S3** & **S4**). At the same time, the effects of dietary patterns and drug dosages were also taken into consideration. Control groups taking Western diets, as well as mice with increased OLA dose, were also tested. The results were consistent with the previous observations (Fig. **S5**, **S6** & **S7**). Taken together, OLA did not increase the risk of obesity in healthy mice.

## 4 DISCUSSION

The molecular mechanisms of OLA regulating adipocyte proliferation and differentiation provide a potential opportunity to explore OLA-induced weight gain and metabolic abnormalities, which may develop possible drug targets and achieve potentially favorable outcomes [13]. To date, no studies have comprehensively explored the molecular mechanisms of OLA-induced weight gain in a multidimensional manner. Thus, there is a pressing need to find molecular biomarkers and key pathways of adipocytes associated with adipocyte proliferation/differentiation. In the present study, we first provided evidence that OLA promotes adipogenesis through direct activation of glycolysis and its downstream PI3K signaling pathway in mature adipocytes. In rodent models, OLA administration increased glycolysis but inhibited the PI3K signaling pathway, although OLA failed to increase food intake and body weight under both short-term and long-term administration.

We found that OLA could directly promote lipid accumulation in both 3T3-L1 cells and AMSCs (Fig. **1**). These findings suggest that OLA treatment may directly alter adipose tissue and influence energy usage, which is consistent with previous reports [30]. *In vivo* and *in vitro* studies have suggested that OLA increases lipogenesis [31, 32]. Notably, we found that OLA had no impact on the differentiation of preadipocytes into mature adipocytes, which was inconsistent with previous studies [31, 33]. The reason may be due to the time-dependent effects or differences in drug dosage. Similar to our findings, it has also been reported that OLA significantly increases lipid droplet accumulation in differentiated human adipocytes but has no effect on adipocyte differentiation [34].

For the first time, our experimental studies provided compelling evidence that OLA affected intracellular glucose uptake through glycolysis and further led to triglyceride storage in adipocytes. Using seahorse, we found that OLA was able to increase the ATP production rate in 3T3-L1 adipocytes (Fig. **2A** & **B**). Furthermore, we inhibited the glycolysis pathway, including the addition of siGAPDH and an inhibitor (VC), and confirmed that glycolysis played an essential role in the process of OLA-induced adipogenesis (Fig. **2D**, **2F**-**2I**). In combination with animal experiments, GAPDH increased after OLA administration in mouse models (Fig. **4G**). GAPDH is located at a branch point between triglyceride metabolism and carbohydrate metabolism. Specifically, GAPDH is crucial for recovering the glycerol released during lipolysis and directing it back into central carbohydrate metabolism as dihydroxyacetone phosphate (DHAP) [35]. In contrast, during triglyceride biosynthesis, DHAP is abstracted from glycolysis, converted to glyceraldehyde 3-phosphate (G3P), and used to form the carbohydrate backbone to which fatty acids are attached [36]. Regarding the action of SGAs on glycolysis, the literature is limited. Similarly, in the rat frontal cortex, it has been reported that OLA treatment has stronger effects on glycolysis/gluconeogenesis after identifying expressed proteins [37]. Glucose transport is rate-limiting for glucose metabolism in muscle and fat, and the impaired insulin-responsive glucose transport system is the key abnormality of glucose intolerance accompanied by insulin resistance [38]. The combination of reduced lipolysis and enhanced insulin anti-lipolysis results in the accumulation of intracellular lipids. This is directly relevant to enlarged adipocyte size and a tendency towards progressive obesity observed in patients treated with these medications.

A further finding of our study was that the PI3K-AKT axis is an essential pathway involved in lipogenesis and glucose uptake. To the best of our knowledge, this is the first finding that olanzapine has led to lipogenesis in adipocytes through glycolysis-dependent PI3K-AKT axis activation. Biochemically, aerobic glycolysis augments ATP and further fuels PI3K-AKT-Foxo1 signaling to generate positive feedback [39, 40]. In addition, we found that OLA induces increased levels of ROS by enhancing glycolysis and, therefore, activates the PI3K-AKT pathway [41, 42] (Fig. **S2**). The PI3K-AKT signaling pathway plays an important role in regulating cellular lipid metabolism [43, 44]. Numerous studies have established a causal relationship between the PI3K-AKT pathway and SREBP, as observed at multiple levels [44, 45]. Previous literature has shown that OLA induces lipogenesis by upregulating SREBP-1 expression [46, 47]. In addition, as a key effector of insulin, Akt decreases glycogen production (*via* GSK3) and increases glucose uptake in muscle cells and adipocytes by stimulating the translocation of the glucose transporter GLUT4 to the plasma membrane [48]. Taken together, insulin resistance might promote lipid synthesis *via* PI3K/Akt/SREBP-related mechanisms.

Notably, in contrast to previous studies [49-51], OLA did not increase weight in different rodent models in our study. To validate the results in mouse models, we tried different intervention durations, dietary patterns (normal diets/western diets) and drug doses in both male and female mice (Fig. **4**, Fig. **S3-S7**). However, in the absence of changes in food intake, the mice showed no significant increase in body weight and abdominal fat or deteriorating glycolipid metabolism. Regarding the impact of OLA administration on weight and metabolism in mice, literature reports have been inconsistent [20, 21]. It is worth noting that OLA failed to activate the downstream PI3K pathway in the mouse model, which could help explain why the WT mice showed no weight gain under OLA administration. In addition, we hypothesize that there are differences in the representativeness of the mouse models for SCZ patients. However, none of the existing animal models of schizophrenia, such as MK801, phencyclidine (PCP), and other drug-induced models, or gene knock-out/editing models (DISC-1, NRG-1 knockouts, Dysbindin-1 mutation) [52], or even combined Bdnf-e6 deficiency and developmental stress models [53], have been able to fully replicate the metabolic profile of first-episode drug-naïve (FEDN) SCZ patients. In previous studies [6, 7, 54, 55], an important caveat is that SCZ patients often exhibit obesity and abnormalities in glycolipid homeostasis before APD treatments. Obese mice display an exaggerated blood glucose response to an OLA challenge [56]. We attempted to investigate the impact of OLA on animal models with impaired insulin resistance (but not yet diabetic or obese), however, the animal model we used did not precisely mimic FEDN SCZ patients. Therefore, the influence of the glucose metabolism state prior to pharmacological intervention on weight change cannot be ignored. In patients with metabolic abnormalities, the impact on metabolism caused by OLA treatment cannot be regulated, further leading to triglyceride storage in body adipocytes and consequently to weight gain. In this regard, further studies are required to fully address the magnitude of short-term OLA-induced weight gain and glycolipid homeostasis in metabolically healthy animals.

SGA-induced weight gain is very complicated and is closely related to insulin resistance, glucose intolerance and lipid metabolism. Interestingly, the changes induced by SGA administration in glucolipid metabolism may, in fact precede weight gain or even no weight gain [57]. Previous literature has reported that acute administration of OLA could directly induce glucose abnormalities by increasing hyperglycemic clamps and decreasing insulin secretion [58, 59]. Blood glucose homeostasis is maintained by the balance between glucose uptake by peripheral tissues and glucose production by the liver [20]. The relationship between these glycolipid metabolic changes and SGA-induced adipogenesis remains to be further elucidated.

Several limitations should be considered when interpreting the above results. Due to the lack of suitable animal models of SCZ or animals with corresponding metabolic characteristics, we were unable to simulate the actual environment in SCZ patients to determine which protective mechanism is defective to make adipocytes more sensitive to OLA treatment. Therefore, in subsequent work, we will perform data collection and index measurements and try to explore the specific metabolic characteristics of first-episode antipsychotic-naive SCZ patients.

## CONCLUSION

**Fig. (5):**
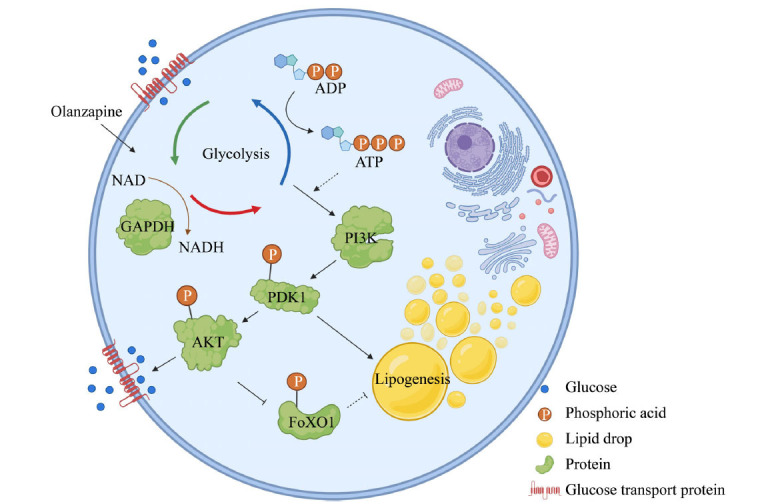
Schematic showing that OLA promotes adipogenesis and glucose uptake by activating glycolysis and synergizing with the PI3K-AKT signaling pathway.

In summary, the present study, along with our previous findings, demonstrates that OLA promotes adipogenesis and glucose uptake by activating glycolysis and synergizing with the PI3K-AKT signaling pathway (Fig. **5**). More importantly, we provide evidence that approaches targeting glycolysis or the PI3K-AKT pathway could be effective interventions to alleviate OLA-induced adipogenesis. Thus, an improved understanding of OLA actions in peripheral tissues may uncover new approaches to improve the long-term safety and utility of this drug.

## Data Availability

The authors confirm that the data supporting the findings of this study are available within the article and its supplementary materials.

## LIST OF ABBREVIATIONS

AIWG: Antipsychotics-induced Weight Gain
AMSCs: Adipose Tissue Mesenchymal Stem Cells
APN: Adiponectin
EF: Epididymal Fat
FEDN: First-episode Drug-naïve
FGAs: First-generation Antipsychotic Drugs
GAPDH: Glyceraldehyde-3-phosphate Dehydrogenase
GLU: Fasting Glucose
HDL: High-density Lipoprotein
LDL: Low-density Lipoprotein
LEP: Leptin
LW: Liver Fat Weight
MetS: Metabolic Syndrome
OLA: Olanzapine
ROS: Reactive Oxygen Species
SCZ: Schizophrenia
SGAs: Second-generation Antipsychotics
TG: Triglycerides
TCHO: Total Cholesterol
