# Supplementary material for: Olanzapine Induces Adipogenesis and Glucose Uptake by Activating Glycolysis and Synergizing with the PI3K-AKT Pathway
Source: Curr Neuropharmacol. 2024 Aug 15;23(4):412–25. doi: 10.2174/1570159X22666240815120547 (PMC12105311; doi:10.2174/1570159X22666240815120547)
Supplement: Supplementary file 1 [file CN-23-4-412_SD1.pdf]

Supplementary Material

Olanzapine Induces Adipogenesis and Glucose Uptake by Activating Glycolysis and Synergizing with the PI3K-AKT Pathway

Shen Li[1,#], Yun Fu[2,3,#], Wanyao Wang[2,&], Jiali Qiu[2,&], Yepei Huang[2], Xuemin Li[2], Ke Yang[2], Xiawen Yu[2], Yanyan Ma[1], Yuan Zhang[2], Miaomiao Zhang[2], Jie Li[1*] and Wei-Dong Li[2,*]

[1]Laboratory of Biological Psychiatry, Institute of Mental Health, Tianjin Anding Hospital, Mental Health Center of Tianjin Medical University, Tianjin, 300222, China; [2]Department of Genetics, College of Basic Medical Sciences, Tianjin Medical University, Tianjin, 300070, China; [3]Fujian Maternity and Child Health Hospital College of Clinical Medicine for Obstetrics and Gynecology and Pediatrics, Fujian Medical University, Fuzhou, 350001, China

1875-6190/25 $65.00+.00 **© 2025 Bentham Science Publishers**

Table S1. Experiment reagents.

Reagent	Company	Cat Number
Penicillin-streptomycin	GIBCO	15070063
Collagenase 1	Sangon Biotech	A004194
Dulbecco's modified eagle medium	Hyclone	SH30022.01
Fetal bovine serum	EveryGreen	11011-8611
Dexamethasone	Solarbio	D8040
Isobutyl-methylxanthine	Solarbio	I8450
Insulin	Sigma	11070-73-8
Olanzapine	MCE	HY14541
Ripa	APPLYGEN	E1013+
Protease inhibitors	APPLYGEN	P1265
PMSF	Solarbio	P0100
SDS-PAGE sample buffer	Genestar	E153
Transference membranes (pvdf)	Merck Millipore	IPVH00010
ECL	Advansta	201005-79
Oil red O	Sigma	O0625
Tissue triglyceride test kit	Comin	XT-1-Y
Seahorse XF Real-Time ATP Rate Assay Kit	Agilent	103677-100
Trypsin solution	Sorfa	BL512A
2-NBDG Glucose Uptake Assay kit (cell-based)	Biovision	K682
Reactive oxygen species assay kit	Beyotime	S0033S
Ascorbic acid	Sigma	A8960
PI3KIN1	MCE	HY-12068
Trizol	Life	260802
cDNA Synthesis Kit	Bimake	B24408
SYBR Green Real-time PCR Master Mix	Bimake	B21703
Adipose tissue protein extraction kit	Biolab	HR0049
High-density lipoprotein cholesterol assay kit	Nanjing jiancheng	A112-1-1
Low-density lipoprotein cholesterol assay kit	Nanjing jiancheng	A113-1-1
Triglyceride assay kit	Nanjing jiancheng	A110-1-1
Glucose kit (hexokinase method)	Nanjing jiancheng	A154-2-1
Total cholesterol assay kit	Nanjing jiancheng	A111-1-1

Table S2. siGAPDH primers.

Name	Sequence
SiGAPDH	Forward: CGAGAAAUAUGACAACUCAdTdT
	Reverse: UGAGUUGUCAUAUUUCUCGdTdT

Table S3. QPCR primers.

Name	Sequence
PPARγ	Forward: TTATGGGTGAAACTCTGGGA
	Reverse: AATCAACTGTGGTAAAGGGC
Adiponectin	Forward: GGACTCTACTACTTCTCTTACC
	Reverse: CAGATGGAGGAGCACAGA
β-actin	Forward: GCACCACACCTTCTACAA
	Reverse: TACGACCAGAGGCATACA
GAPDH	Forward: TCTCCTGCGACTTCAACA
	Reverse: TGTAGCCGTATTCATTGTCA
RAC1	Forward: TGTAGCCGTATTCATTGTCA
	Reverse: GTCGCACTTCAGGATACC
FAS	Forward: GCCCGGTAGCTCTGGGTGTA
	Reverse: TGCTCCCAGCTGCAGGC

Table S4. Grouping of rodent models.

Group	Model		Dose (mg/kg*d)	Sex	Food Type	Time (Week/Month)	Number of Mice
Group1	A1	C57BL6/J	0	Male	M	2w	9
	A2	C57BL6/J	3	Male	M	2 w	9
Group2	B1	C57BL6/J	0	Female	M	2 w	9
	B2	C57BL6/J	3	Female	M	2 w	9
Group3	C1	C57BL6/J	0	Male	W	2 w	10
	C2	C57BL6/J	3	Male	W	2 w	9
Group4	D1	C57BL6/J	0	Female	W	2 w	9
	D2	C57BL6/J	3	Female	W	2 w	9
Group5	E1	C57BL6/J	0	Male	M	6 m	10
	E2	C57BL6/J	3	Male	M	6 m	10
Group6	F1	SD	0	Male	M	2 m	5
	F2	SD	3	Male	M	2 m	5
Group7	G1	SD	0	Male	W	2 m	5
	G2	SD	3	Male	W	2 m	5
Group8	H1	KK/Upj-Ay	0	Male	W	3 m	6
	H2	KK/Upj-Ay	3	Male	W	3 m	6
Group9	I1	C57BL6/J	0	Male	W	3 m	5
	12	C57BL6/J	16	Male	W	3 m	5

SD: Sprague Dawley Rats; M: maintenance feed; W: Western diet feed.

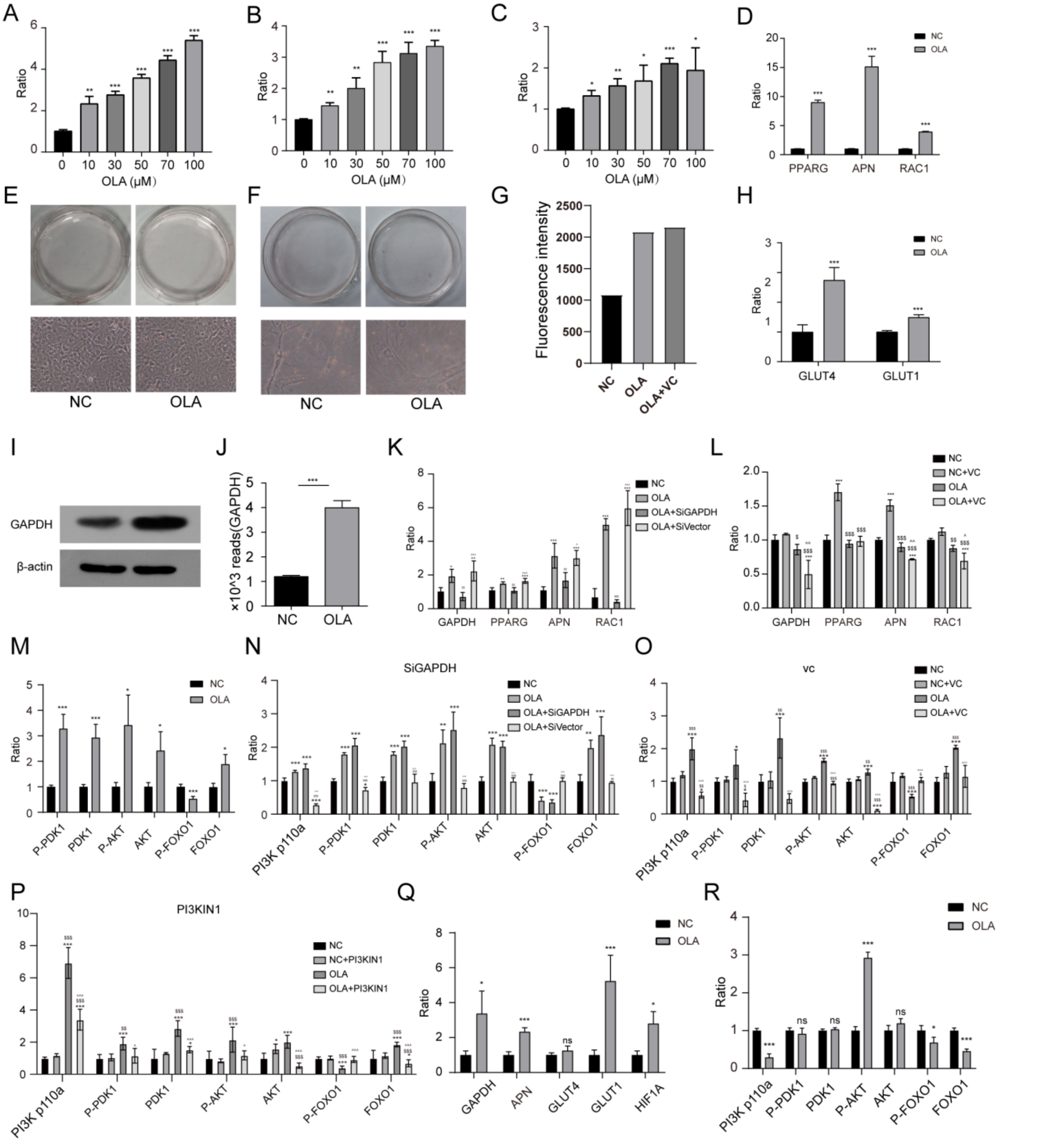

Fig. (S1). Effects of OLA on adipogenesis, glycolysis pathway, and PI3K pathway in undifferentiated preadipocytes. (**A-C**) Western blot quantification of PPARG, APN, and RAC1 for Figure **1E**, normalized by β-actin; (**D**) Western blot quantification of PPARG, APN, and RAC1 for Figure **1G**, normalized by β-actin; (**E**) Images of Oil Red-O (ORO) staining of undifferentiated 3T3-L1. Olanzapine (OLA) did not promote lipid accumulation in undifferentiated 3T3-L1 cells; (**F**) ORO staining of undifferentiated AMSCs; (**G**) Quantification of glucose uptake under fluorescence intensity for Figure 2E. (**H**) Quantitative analysis of GLUT1 and GLUT4 expressions for Figure **2G**; (**I**) Western blot of GAPDH in differentiated 3T3-L1 (N=3); (**J**) Transcription level of GAPDH in differentiated 3T3-L1 cells. (K) Western blot quantification of GAPDH, PPARG, APN, and RAC1 for Figure **2H**, normalized by β-actin (N=3); (**L**) Western blot quantification of GAPDH, PPARG, APN, and RAC1 for Figure **2J**, normalized by β-actin (N=3); (**M**) Quantitative analysis of PI3K pathway gene expression and phosphorylation levels for figure 3B (N=3); (**N**) Quantitative analysis of PI3K pathway gene expression and phosphorylation levels for Figure **3F** left (N=3); (**O**) Quantitative analysis of PI3K pathway gene expression and phosphorylation levels for Figure 3F middle (N=3); (**P**) Quantitative analysis of PI3K pathway gene expression and phosphorylation levels for Figure **3F** right (N=3); (**Q**) Quantitative analysis of glycolysis related gene expression levels for figure 4G (N=3). (**R**) Quantitative analysis of PI3K pathway gene expression and phosphorylation levels for Figure 4H (N=3). Values are expressed as mean ± SD, $^{*}P < 0.05$, $^{**}P < 0.01$, $^{***}P < 0.001$.

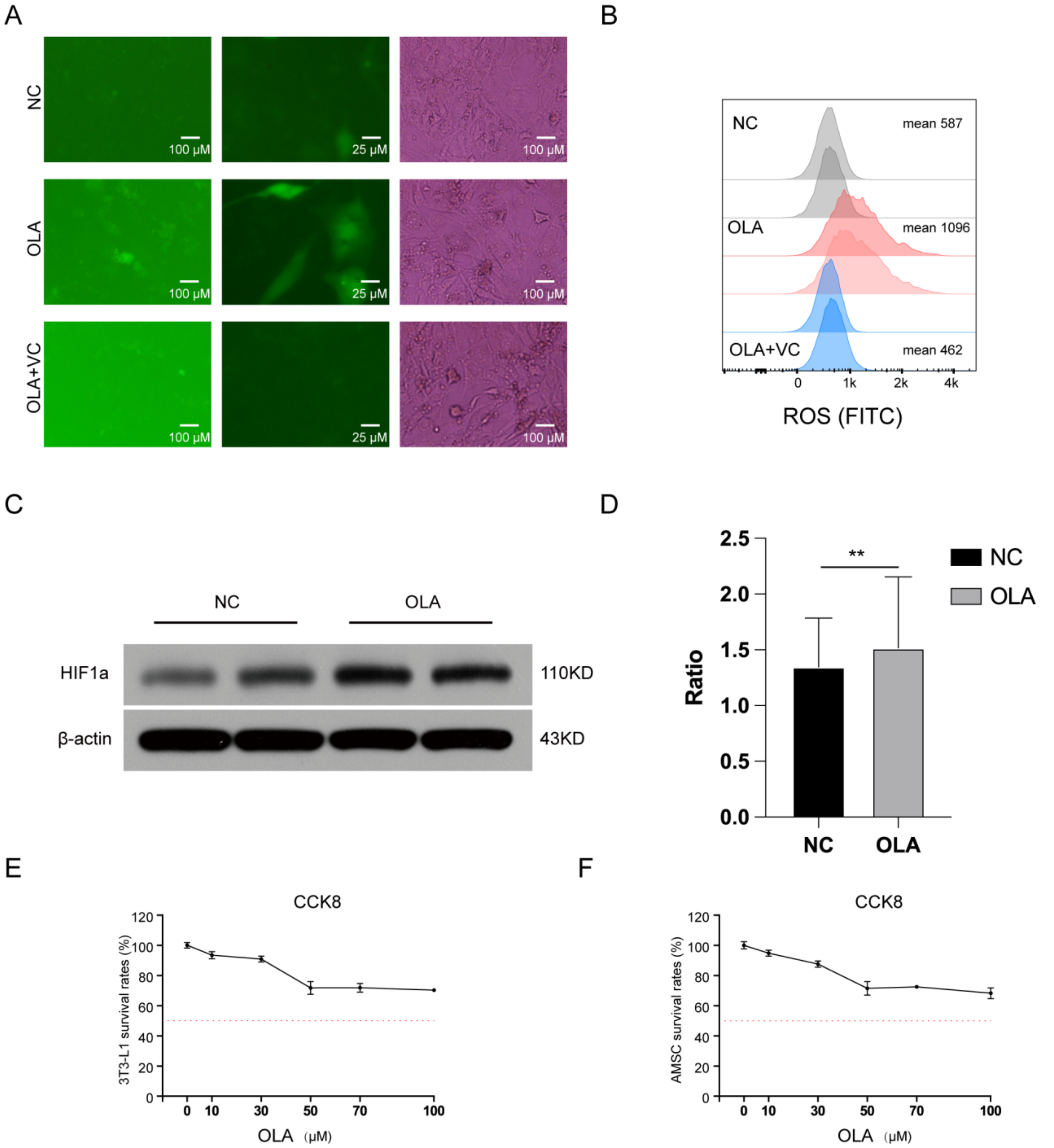

Fig. (S2). ROS productions in OLA treated 3T3L1 preadipocytes. (**A**) ROS expression level after DMSO, OLA treatment and VC treatment under fluorescence microscope. OLA raised intracellular ROS and alleviated by VC. (**B**) ROS expression level after DMSO, OLA treatment and VC treatment under flow cytometer. (**C**) Western blot of HIF1 alpha in differentiated 3T3-L1 (N=3); (**D**) Western blot quantification of HIF1A for Figure **S2C**, normalized by β-actin (N=3); (**E**)&(**F**) Dose response curves for olanzapine in 3T3-L1 (**E**) and AMSC by CCK8-assays.

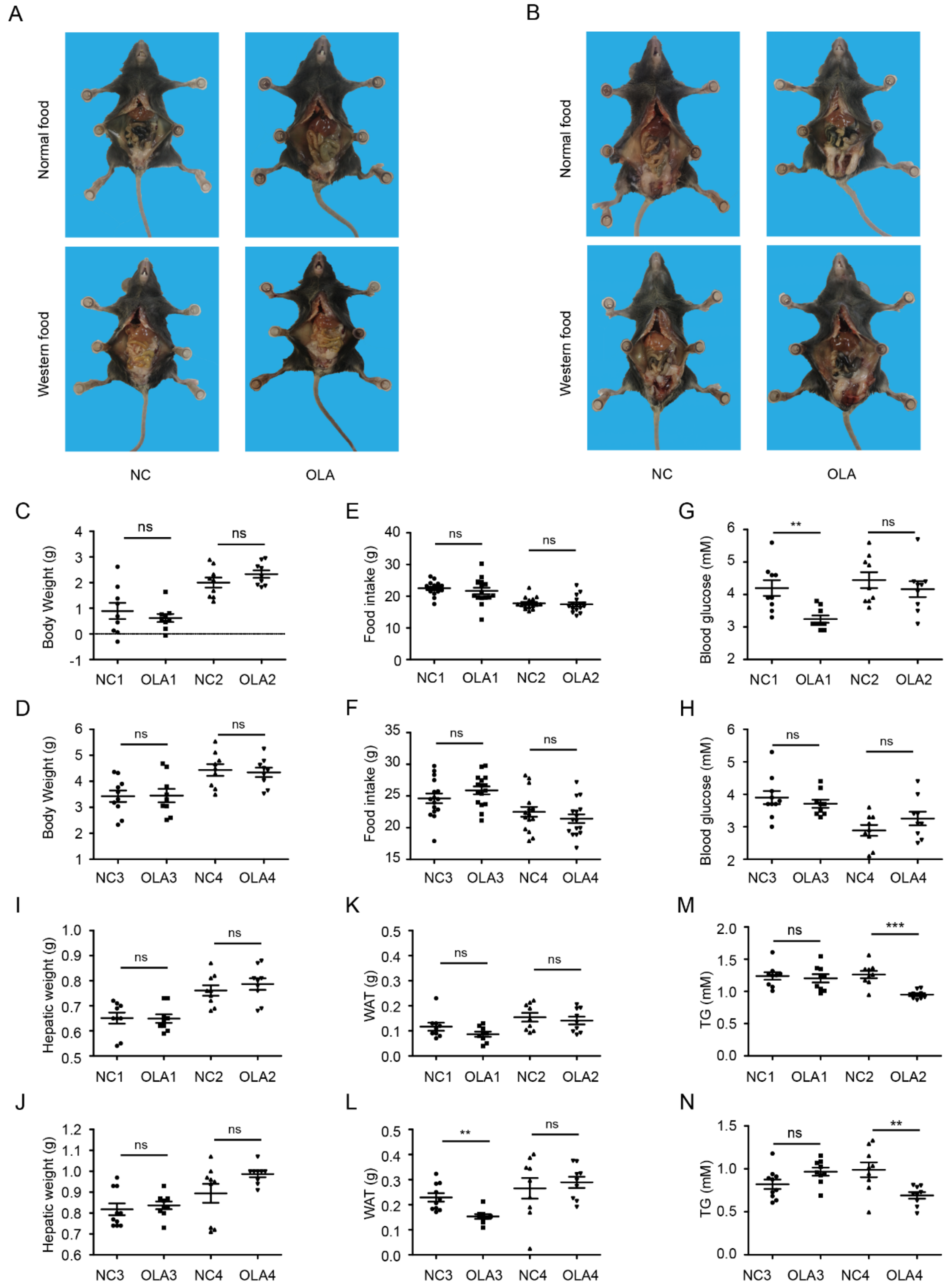

Fig. (S3). After two weeks of OLA exposure, female and male C57BL/6J mice on normal and Western diets did not experience weight gain or metabolic disorders. (**A**) female mouse anatomy, upper left is normal food intake control group (with saline) upper right is normal food intake OLA treated group (with OLA, 3mg/kg/day). Lower left is western food intake control group (with saline), lower right is Western food intake OLA treated group (with OLA, 3mg/kg/day). (**B**) male mouse anatomy, upper left is normal food intake control group (with saline) upper right is normal food intake OLA treated group (with OLA, 3mg/kg/day). Lower left is western food intake control group (with saline), lower right is Western food intake OLA treated group (with OLA, 3mg/kg/day). Two weeks of OLA treatment did not result in an increase in body fat in female and male mice on normal or Western diets. (**A**)&(**B**) body weight; (**E**)&(**F**) food intake per day; (**G**)&(**H**) fasting glucose; (**I**)&(**J**) hepatic weight; (**K**)&(**L**) fat around the epididymis or ovary; (**M**)&(**N**) blood triglycerides (TG). Values are expressed as mean ± SD, $*P < 0.05$, $**P < 0.01$, $***P < 0.001$, ns, no significant difference, N=9.

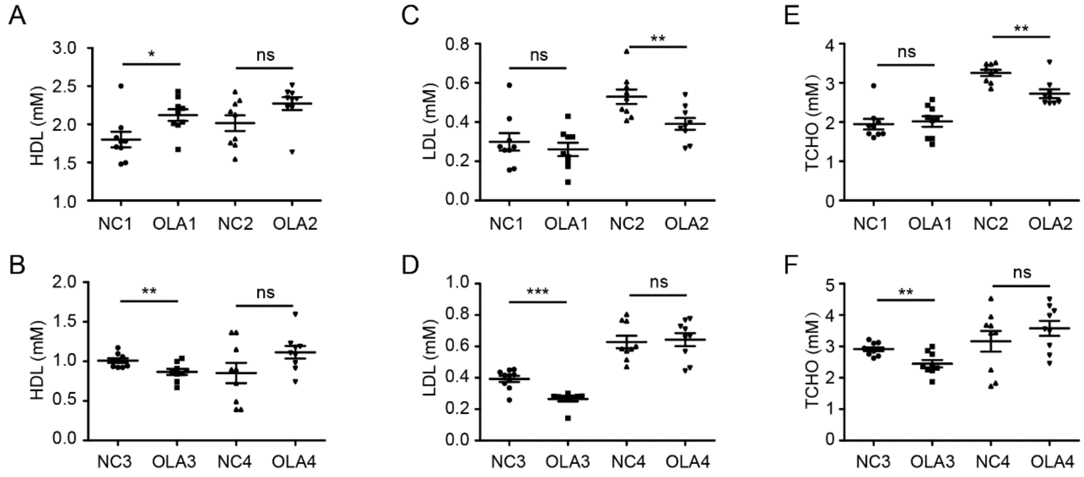

Fig. (S4). After two weeks of OLA exposure, female and male C57BL/6J mice on normal or Western diets did not experience hyperlipidemia. (**A**)&(**B**) serum high density lipoprotein (HDL); (**C**)&(**D**) serum high density lipoprotein (LDL); (**E**)&(**F**) serum total cholesterol (TCHO). Values are expressed as mean ± SD, **P* < 0.05, ***P* < 0.01, ****P* < 0.001, ns, no significant difference, N=9.

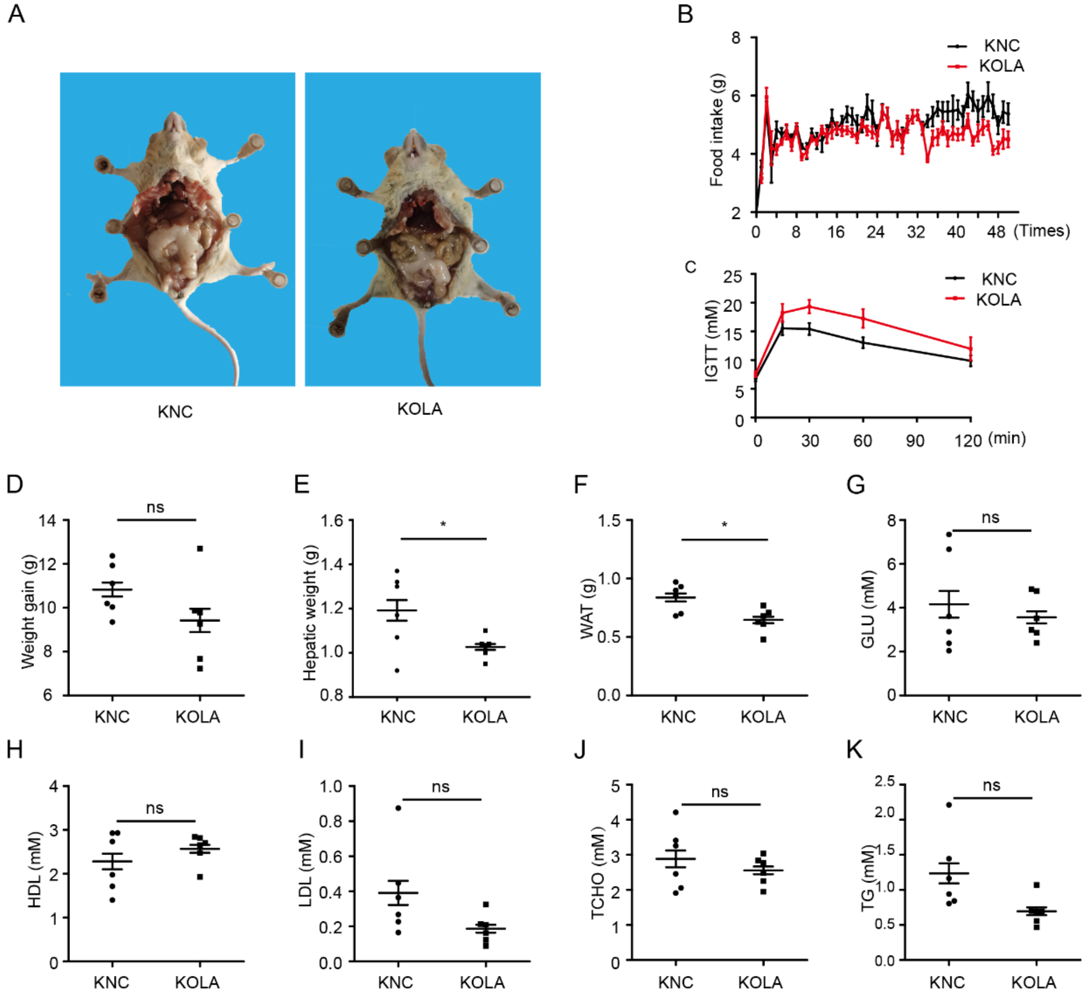

Fig. (S5). Male KK-ay mice exposed to OLA for three months did not have weight gain or metabolic disorders. (**A**) mouse anatomy. left is control group (with saline). right is OLA treated group (with OLA, 3mg/kg/day); (**B**) food intake (every 4 days); (**C**) IGTT (intraperitoneal glucose tolerance test); (**D**) weight gain; (**E**) hepatic weights; (**F**) fat around the epididymis (WAT); (**G**) fasting glucose; (**H**) serum high density lipoprotein (HDL); (**I**) serum high density lipoprotein (LDL); (**J**) serum total cholesterol (TCHO); (**K**) blood triglycerides (TG). Values are expressed as mean ± SD, **P* < 0.05, ***P* < 0.01, ****P* < 0.001, ns, no significant difference, N=6.

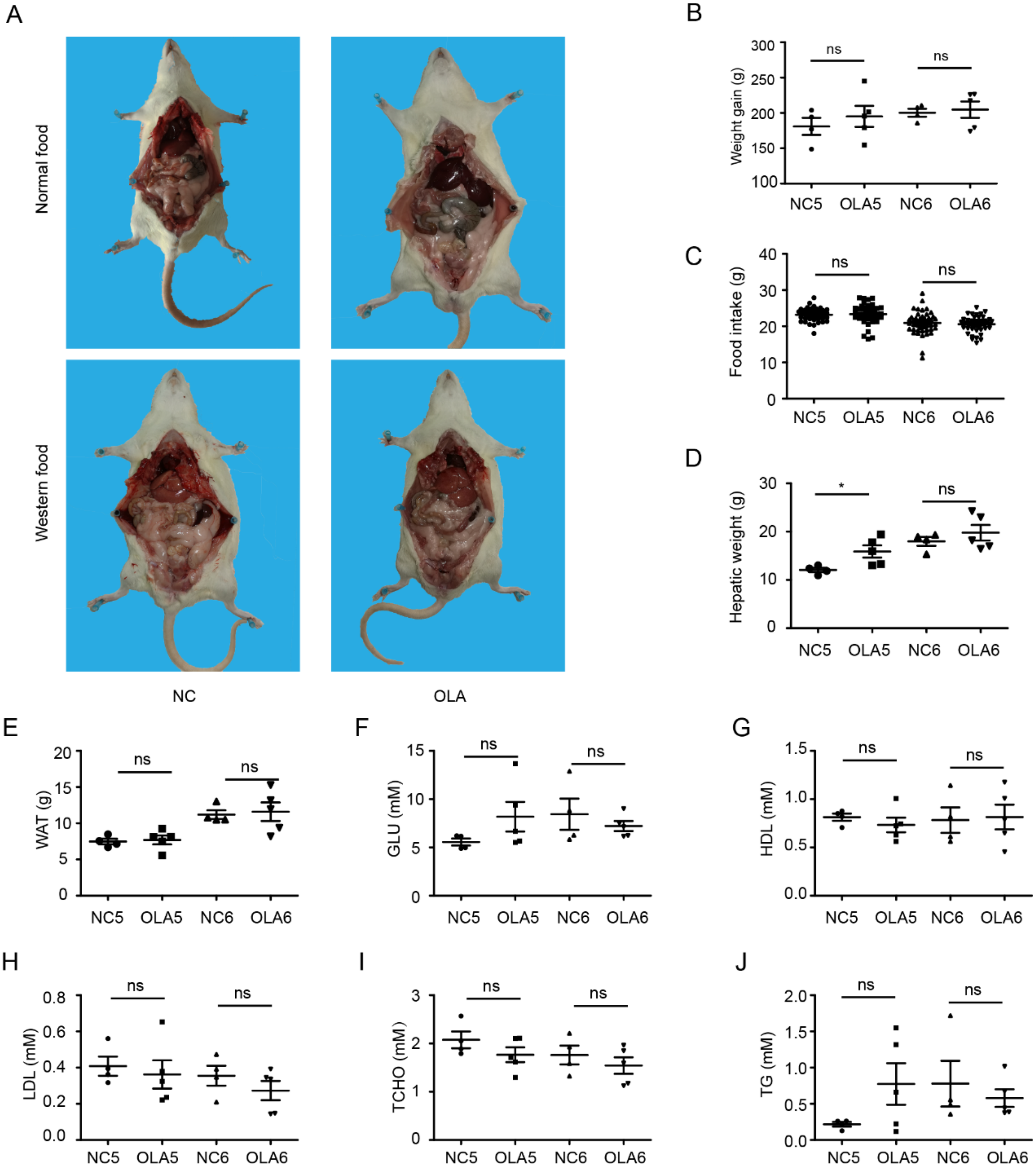

Fig. (S6). After two months of OLA exposure, male Sprague Dawley rats on normal and Western diets did not experience weight gain or metabolic disorders. (**A**) rat anatomy, upper left is the normal food intake control group (with saline) upper right is normal food intake OLA treated group (with OLA, 3mg/kg/day). Lower left is Western group (with OLA, 3mg/kg/day); (**B**) body weight gain; (**C**) food intake (every 4 days); (**D**) hepatic weights; (**E**) fat around the epididymis (WAT); (**F**) fasting glucose; (**G**) serum high density lipoprotein (HDL); (**H**) serum high density lipoprotein (LDL); (**I**) serum total cholesterol (TCHO); (**J**) blood triglycerides (TG). Values are expressed as mean ± SD, $*P < 0.05$, $**P < 0.01$, $***P < 0.001$, ns, no significant difference, N=5.

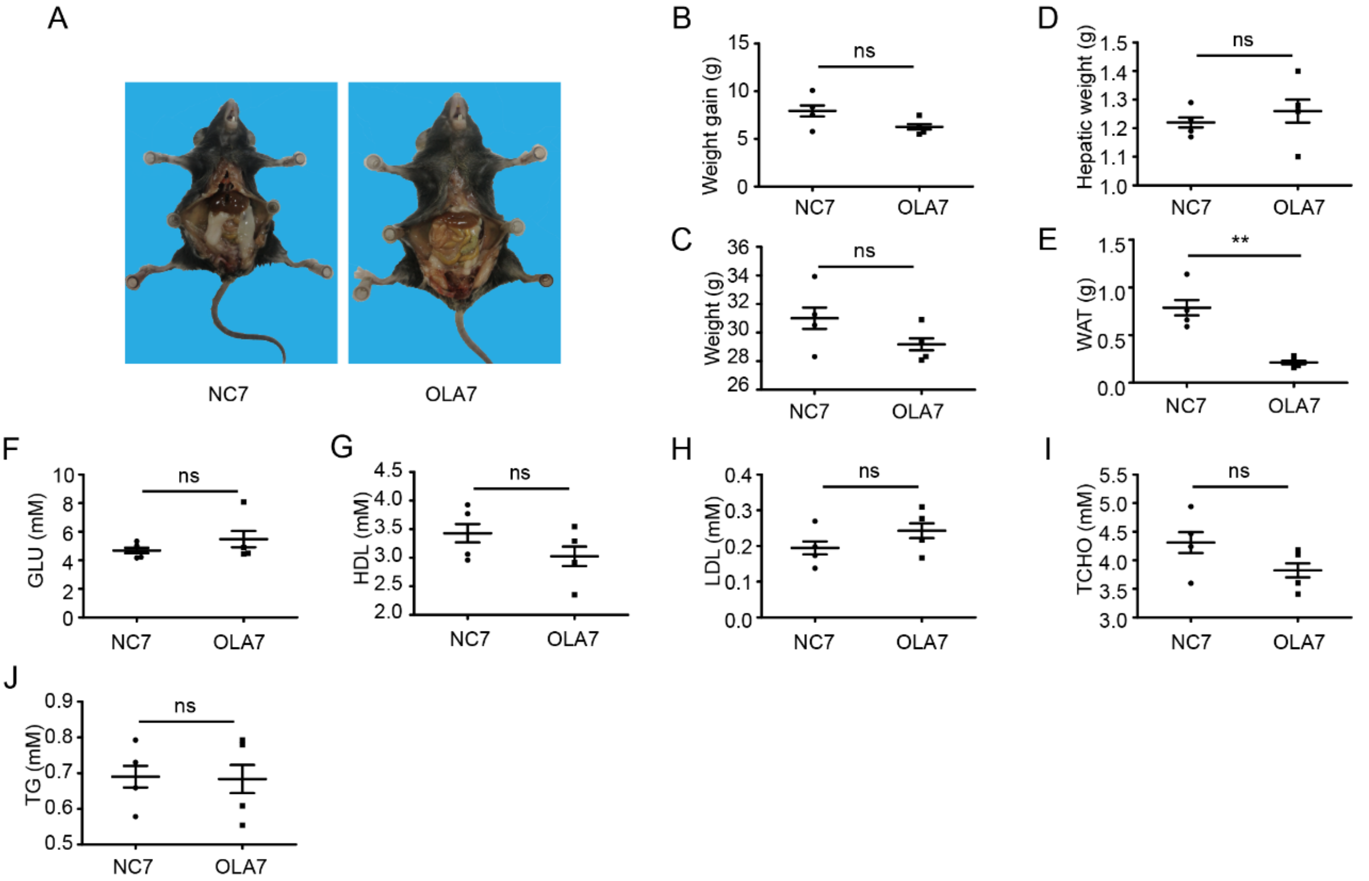

Fig. (S7). After three months of OLA exposure, male C57BL/6J mice on Western diets did not experience weight gain or metabolic disorders. (**A**) mouse anatomy, left is normal food intake control group (with saline), right is OLA treated group (with OLA, 16mg/kg/day); (**B**) body weight gains; (**C**) body weights; (**D**) hepatic weights; (**E**) fat around the epididymis (WAT); (**F**) fasting glucose; (**G**) serum high density lipoprotein (HDL); (**H**) serum high density lipoprotein (LDL); (**I**) Serum total cholesterol (TCHO); (**J**) blood triglycerides (TG). Values are expressed as mean ± SD, **P* < 0.05, ***P* < 0.01, ****P* < 0.001, ns, no significant difference, N=4.
